# The role of particle therapy in the treatment of locally recurrent rectal cancer: a systematic review

**DOI:** 10.2478/raon-2026-0036

**Published:** 2026-09-07

**Authors:** Ana Perpar, Petra Georg, Ajra Secerov Ermenc

**Affiliations:** 1 Department of Radiation Oncology, Institute of Oncology Ljubljana, Ljubljana, Slovenia; 2 Department of Radiation Oncology, University Clinic Krems, Krems an der Donau, Austria; 3 Karl Landsteiner University of Health Sciences, Krems an der Donau, Austria; 4 Medical Faculty, University of Ljubljana, Ljubljana, Slovenia

**Keywords:** rectal cancer, inoperable local recurrence, unresectable recurrence, particle therapy, ion beam therapy, SBRT, curative RT

## Abstract

**Background:**

Colorectal cancer is a common malignancy. Advancements in multimodality treatment have improved outcomes. About 2–10% of patients will have a local recurrence even after optimal treatment. Salvage surgical treatment is the treatment of choice, however, in posterior and lateral recurrences, surgery is linked to a high rate of treatment-related morbidity. Some recurrences remain unresectable even after neoadjuvant treatment.

**Materials and methods:**

A systematic literature review was performed to search for studies on curative-intent radiation therapy (RT) with photons, stereotactic body radiation therapy (SBRT) and particle beam therapy. The aim of the literature search was to define the role of particle therapy in the treatment of patients with unresectable or inoperable local recurrences of rectal cancer where neoadjuvant treatment is unlikely to result in downstaging, leaving patients with RT as the only curative treatment option.

**Results:**

32 studies which fulfil the criteria were identified. In general, SBRT and particle beam therapy permitted the application of significantly higher doses to the target without a concomitant increase in treatment-related toxicities.

**Conclusions:**

For unresectable and inoperable locally recurrent rectal cancer ablative radiotherapy techniques such as SBRT and particle beam therapy offer a curative treatment approach as an alternative to surgery. SBRT can be offered for small local and nodal recurrences, while particle beam therapy can be offered for larger recurrences and complex shapes spanning several anatomical compartments as well. Further research is needed to stratify patients according to their need and eligibility for the different possible modalities of curative-intent RT.

## Introduction

Colorectal cancer is the third commonest cancer in men and the second commonest cancer in women in the European Union.^1^ With improvements in treatment, e.g. the use of total mesorectal excision in surgical treatment, the use of neoadjuvant and later total neoadjuvant treatment combining radiotherapy and systemic treatment, including immunotherapy, the risk for local and pelvic recurrence has been significantly reduced, the 5-year followup of the RAPIDO trial yielded a 10% rate of locoregional failure in the experimental group and may be as low as 2%, according to some results.^2-9^ However, for patients experiencing a pelvic recurrence, curative treatment options are limited.

Rokan *et al*. have published a review grouping local recurrences according to anatomical site of recurrence: central (18%), lateral (15%), posterior (13%), anterior below peritoneal reflection (10%), infralevator/anterior urogenital triangle (4%), and anterior above peritoneal reflection (1%), unknown 39%.^10^

The preferred treatment for pelvic recurrences is salvage surgery which must be undertaken with the aim of achieving negative margins, since complete (R0) resections have been consistently linked to significantly better survival outcomes than nonradical resections.^11-13^

Unfortunately, not all sites of recurrence have the same resectability and prognosis. Central recurrences have consistently been shown to result in the highest rate of R0 resection, lateral and posterior recurrences are the least likely to be successfully treated with an R0 resection and even worse results have been reported for recurrences involving more than one compartment.^12,14-17^

Additionally, aggressive surgical treatment of lateral and posterior recurrences results in a higher rate of severe impairments, including severe neurological dysfunction of bladder and bowel in high sacral resection, while extensive lateral resection is linked to a 82% rate of postoperative complications.^18^ And though exenteration may offer patients with locally advanced rectal cancer the chance of long-term survival with acceptable levels of morbidity, it remains a major operation associated with significant morbidity and mortality where the R1 resection rate remains high, especially in recurrent cancers (median 28.26%, upper range 68.2%).^19,20^

The use of neoadjuvant chemoradiation in locally recurrent rectal cancer is recommended by both ESMO and Beyond TME guidelines, with the aim of downstaging and improving resectability.^21,22^ In patients who had already received a radiation course to the pelvis, the prescribed dose must be individualized to reach a compromise between maximum safe dose and the dose constraints of pelvic organs. The Dutch national guidelines for the management of locally recurrent rectal cancer report the use of intensity-modulated radiation therapy / volumetric modulated arc therapy (IMRT/VMAT) use as the standard of care in the Netherlands both for primary and reirradiation of locally recurrent rectal cancer.^23^

There is however a significant proportion of patients with locally recurrent rectal cancer who either remain unresectable after neoadjuvant treatment or are not candidates for surgical treatment due to other reasons (e.g. the patients’ poor general condition, comorbidities or refusal to consent to treatment which may leave them with life-long functional compromise). If lateral and posterior recurrences from Rokan *et al*. are grouped together, we have over a quarter (28%) of all local recurrences with a significant likelihood of unresectable disease even after neoadjuvant treatment.^10^

There is evidence that local recurrences after radiation therapy shift from central and anterior to more lateral and posterior.^24^ This means that an even larger proportion of patients who received neoadjuvant radiation as part of their primary treatment have recurrences in unfavourable locations.

For these patients, curative-intent radiation therapy (RT) presents the only chance of cure. The development of modern treatment techniques – stereotactic body radiation therapy (SBRT) and intensity-modulated radiation therapy (IMRT) – as well as the use of charged particles with a more favourable dose distribution profile makes the application of a curative dose possible.

The risk of severe radiation-induced toxicity depends on where the recurrence is located. In posterior and lateral recurrences, the major risks are radiation-induced damage to the rectum (with possible haemorrhage, perforation and fistulas) and neurotoxicity (with sensorimotor impairments and neuropathic pain), while high-dose radiation therapy of central and anterior recurrences, while still linked with increased risk of rectal damage, also carries the risk of bladder toxicity (haemorrhage, perforation and fistulas). Any recurrence abutting the bony structures of the pelvis also increases the risk of bone fractures.

For the purposes of this article, we have focused on unresectable or inoperable local recurrences of rectal cancer where neoadjuvant treatment is unlikely to result in any significant downstaging, leaving patients with definitive RT as the only curative treatment option. In this narrative review we wish to summarize the current state of the art in the treatment of these patients and establish the role of particle therapy in their treatment of patients.

## Materials and methods

This review was conducted using the Preferred Reporting Items for Systematic Reviews and Meta-Analyses (PRISMA) guidelines.^25^ Eligible sources included published and peer-reviewed work evaluating

(1) clinical outcomes (including local control and overall survival was mandatory, data on toxicities was desired, but not necessary for inclusion) of

(2) conformal photon, SBRT or particle beam therapy with curative intent for

(3) pelvic recurrences of rectal cancer in

(4) both radiation-naïve and pre-irradiated patients, reported in

(5) English with

(6) a minimum number of 10 reported cases. Studies had to report on the

(7) radiation dose applied,

(8) any concomitant or additional treatment the patients received, e.g. concomitant chemotherapy, hyperthermia or surgery,

(9) pelvic control outcomes (including local and regional control, if reported) and

(10) survival.

We have searched PUBMED, EMBASE, SCOPUS, Clinicaltrials.gov and COCHRANE, those found in references from the major articles identified, and articles known to the authors. To avoid missing potentially relevant publications, sources were excluded by individual screening of reports rather than screening of titles and abstracts. We have also scanned the references of included articles for missed publications.

The first search was conducted in December 2023, then updated in September / October 2024.

An initial screening was performed using citations and titles, to filter studies duplicated among databases or studies that were not clinical trials, such as reviews, letters, editorials, and in vivo or in vitro studies. A second screening of abstracts and excluded studies with irrelevant subjects, fewer than 10 patients with rectal cancer receiving reirradiation, and studies that were not clinical trials was conducted. Finally, a full-text review to identify studies meeting the above inclusion criteria was performed. In cases of multiple studies from a single institution, the following criteria were applied and prioritized in the following order: (1) studies with the largest number of patients with rectal cancer who received reirradiation, and (2) the most recent publication. Even if studies were published by the same institution, multiple studies were included if the patient cohorts could safely be assumed to be different.

Only articles that report patient outcomes on the usual timepoints were included, i.e. full years. The report by Habermehl *et al*. was excluded for this reason.^26^

Studies on conventional photon radiation therapy were excluded if they reported results on older treatment techniques used before the adoption of 3-dimensional conformal radiation therapy (3D-CRT), or if no definite information was available in the article and it could safely be assumed the cohort was treated with older techniques.

Studies reporting on locally recurrent rectal cancer amongst other pelvic malignancies (e.g. together with locally advanced rectal cancer or together with recurrent urological and/or gynaecological malignancies) were included only if the results of treatment of locally recurrent rectal cancer were reported separately from the other entities and if the number of the included patients with locally recurrent rectal cancer was 10 or more. Moningi *et al*. and Shirai *et al*. were excluded for this reason.^27,28^

The above screening processes was performed by AP and checked by PG & AŠE, final inclusion was confirmed upon mutual consent.

The following terms were used:

**Table:** 

((carbon[Title/Abstract]) OR (hadron[Title/Abstract]) OR (hadrons[Title/Abstract]) OR (carbon ion beam[Title/Abstract]) OR (carbon ion[Title/Abstract]) OR (proton[Title/Abstract] OR (protons[Title/Abstract]) OR (proton beam[Title/Abstract]) OR (charged particle[Title/Abstract]) OR (charged particles[Title/Abstract]) OR (particle[Title/Abstract]))	OR	((SBRT[Title/Abstract]) OR stereotactic[Title/Abstract]) OR stereotaxy[Title/Abstract]))	OR	((radiation[Title/Abstract]) OR radiotherapy[Title/Abstract]) OR (radiation therapy[Title/Abstract]) OR (reirradiation[Title/Abstract]) OR (irradiation[Title/Abstract]) OR (actinotherapy[Title/Abstract]))
AND				
(rectal[Title/Abstract] OR (rectum[Title/Abstract]))				
AND				
((recurrence[Title/Abstract]) OR (relapse[Title/Abstract]) OR (local recurrence[Title/Abstract]) OR (pelvic recurrence[Title/Abstract]) OR (pelvic relapse[Title/Abstract]) OR (local relapse[Title/Abstract]) OR (locally recurrent[Title/Abstract]) OR (recurrent[Title/Abstract]) OR (pelvis[Title/Abstract]) OR (pelvic[Title/Abstract]) OR (pelvic[Title/Abstract]))				

SBRT = stereotactic body radiation therapy

For Clinicaltrials.gov the terms were: rectal and rectum for condition/disease and particle, carbon or proton for other terms.

A search for currently active studies on radiation therapy of locally recurrent rectal cancer was performed.

## Results

**FIGURE 1.**
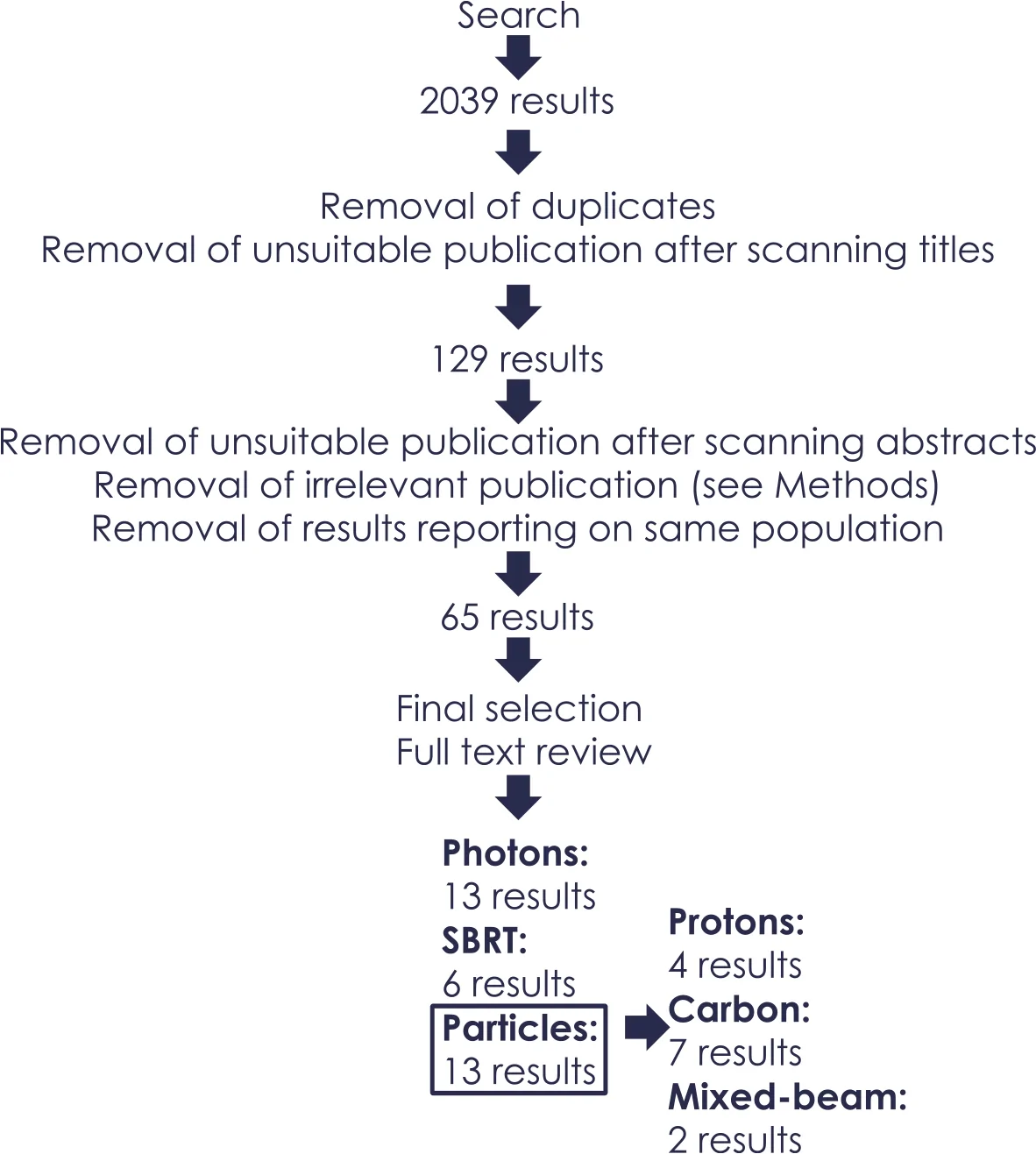
Process of article selection

32 studies which fulfil the criteria were identified, 12 studies reporting on the results of photon RT, 6 studies reporting on the results of SBRT, 13 studies reporting on the results of particle beam therapy. The process of selection is depicted in Figure 1.

### Multimodality treatment

Several studies have reported improved outcomes in patients treated with multimodality therapy and in which an R0 resection could be achieved.^29,30^

The results of photon RT are presented in Supplementary Table 1.^31-42^

Surgery after radiation has been shown to result in superior outcomes, including local control as well as survival, in addition, tumour size and total dose applied were predictive of improved outcomes. However, in some reports, surgery as well as higher applied dose have also been linked with a higher risk of severe late toxicity.^31,34,36,39-41,43-45^

Patients unlikely to have resectable disease even after neoadjuvant chemo(re)irradiation are unlikely to benefit from surgical treatment and for them, the outcomes for definitive radiation therapy with photons remain dismal, please see Supplementary Table 1 for further details. These patients urgently require a treatment alternative, since for most of them, their survival is determined by local control of the recurrence.^31,39,41^

Numerous strategies to enhance the therapeutic ratio have been explored, including concomitant systemic treatment, alterative fractionation regimes, to the use of intraoperative RT which, owing to the physical characteristics of the beams used, electrons, kilovoltage photons, or brachytherapy, can facilitate the application of a higher cumulative dose without significantly increasing the risk for late toxicities.^31-33,36,39,41-43,46,47^ Photons, given their physical limitations, cannot apply a curative dose to the recurrent tumour outside of stereotactic radiation modalities and are used in a neoadjuvant setting or a means of palliation.

It should also be pointed out that several of the studies included patients with metastatic disease, which undoubtedly had an impact on overall survival. In the studies reporting on the results of definitive RT, only Cai *et al*. report including patients with metastatic disease.^31,33,34,36,40,41^

### Definitive radiation therapy

The results of definitive RT with SBRT or particle beams are presented in Supplementary Table 2.^48-66^

### Stereotactic body radiation therapy (SBRT)

The included studies report clinical results for various follow-up intervals; thus an esti mate of expected local control is difficult. In addition, dose prescription regimes within individual publications are highly variable as most publications report only a mean dose or biologically equivalent dose (BED) or alternatively a range of doses and fractions.^48,49,51,52^ The results of a uniform dose prescription protocol (30 Gy in 5 fractions) are reported only by Johnstone *et al*. and Smith *et al*.^50,53^

2-year local control can be expected to be 68.2–93.7%, while 2-year survival was reported to be 65.9-84.4%.^48-50,53^ Minecci *et al*.^52^ report 5-year local control rates of 82.6%, Kim *et al*. report 5 year-survival 23.2%.^51,^ The incidence of late grade 3 or more toxicities ranges from 0–13%.^49,52^

In the studies reported, the median tumour size ranges from 13.4 to 90.1 cm^3^ or up to 136.5 cm^3^ for the median planning target volume (PTV) volume.^48,50,52^ The vast majority of treated patients were treated for pelvic nodal rectal cancer recurrences, which confirms that SBRT is the radiation modality of choice for delivering high dose to a smaller volume of relatively simple shape.

### Particle beam therapy

The usual dose regimes used in the Japanese centres for carbon ion beam therapy consists of 16 fractions with a total dose of 67.2–73.6 Gy. 5-year local control rates are reported to be between 35–88% with a clear dose-response relationship, which is also mirrored in the reported 5-year overall survival.^54^

Other centres using carbon ion beams have more heterogeneous dose prescription, however Cai *et al*. have also observed a dose-response relationship with the cutoff at 66 Gy.^64^

It is difficult to compare the results of proton beam therapy since the publications we have included report outcomes at different time intervals and do not use uniform dose prescription regimes, however, 1-year and 3-year local control is reported as between 66.3-70% and 55-80.2%.^56,57,60,62^

The volumes of treated targets are variable, but from the studies that report volumes of targets, the median gross target volume (GTV) volume ranges from 57.1–154 cm^3^ for carbon ions and 26.1-86.4 cm^3^ for protons, with maximum volumes over 300 cm^3^. Others report only median target diameters which range from 30–63 mm.^56,58,60,65,66^

In RT-naïve patients, the toxicity profile is very favourable and in general late toxicities of particle beam therapy grade 3 or higher are reported in up to 5% of cases, with rare grade 4 toxicities, though Takagawa *et al*. report grade 4 gastrointestinal toxicities in 3 patients in 2 of whom reirradiation was associated with further local recurrence after proton beam therapy.^54,57,62,67,68^ In patients treated with reirradiation severe toxicities are reported more frequently, up to 21%.^55^ There is also mention of grade 5 toxicities, in particular Koroulakis *et al*. report a fatal presacral haemorrhage in patient previously treated with radiation for ovarian cancer in the 1970s who developed a grade 4 presacral skin ulcer.^60^

### Clinical trials

The ongoing studies are summarized in Supplementary Table 3.^69-77^

There are several studies currently recruiting patients with locally recurrent rectal cancer for particle beam RT.

Combs *et. al* published the protocol of the PANDORA-01 study in 2012^78^, describing the protocol for their prospective phase I/II study for carbon ion beam therapy for recurrent rectal cancer. As of now, no clinical results have been published.

## Discussion

Since the majority of the included studies were retrospective, non-controlled studies, there is an inherent risk of bias in our publication.

As is evident from this review, patients with locally recurrent rectal cancer who do not receive a complete resection have dismal outcomes after conformal photon radiation therapy. However, both SBRT as well as particle therapy offer a curative non-invasive treatment option for these patients, both RT-naïve and preirradiated. Both SBRT as well as particle beam therapy allow for the application of ablative doses to the target in contrast to conformal photon RT. The application of sufficient dose in the context of ablative radiation therapy is crucial to the curative intent.^61,63,64^ Yamada *et al*. have demonstrated that raising the prescribed dose from 67.2 Gy relative biological effectiveness (RBE) to 73.6 Gy RBE more than doubles both 5-year local control as well as 5-year overall survival.^54^

The main limiting factors to the use of SBRT are target size and shape; while small local and nodal recurrences are good candidates for SBRT, larger recurrences with complex shapes spanning several anatomical compartments are not optimal candidates for SBRT. The definitions of SBRT treatment almost never include the maximum size of the target, specifying only that an ablative dose must be safely applied to the target, the commonly used cut-off for lung tumours is 5 cm (with acceptable results published for larger tumours) and 6 cm for liver tumours, both of which usually have simple, circular shapes.^79,80^ Any attempt at generalizing these results to tumours with complex concave shapes is difficult if not impossible. In our review, studies that reported the range of tumour volumes reported maxima under 150 cm^3^, with Dagoglu *et al*. as the only exception, where the maximum size of lesions was over 1 litre, with the median under 100 cm^3^, meaning that most targets were still relatively small.^48^

**FIGURE 2.**
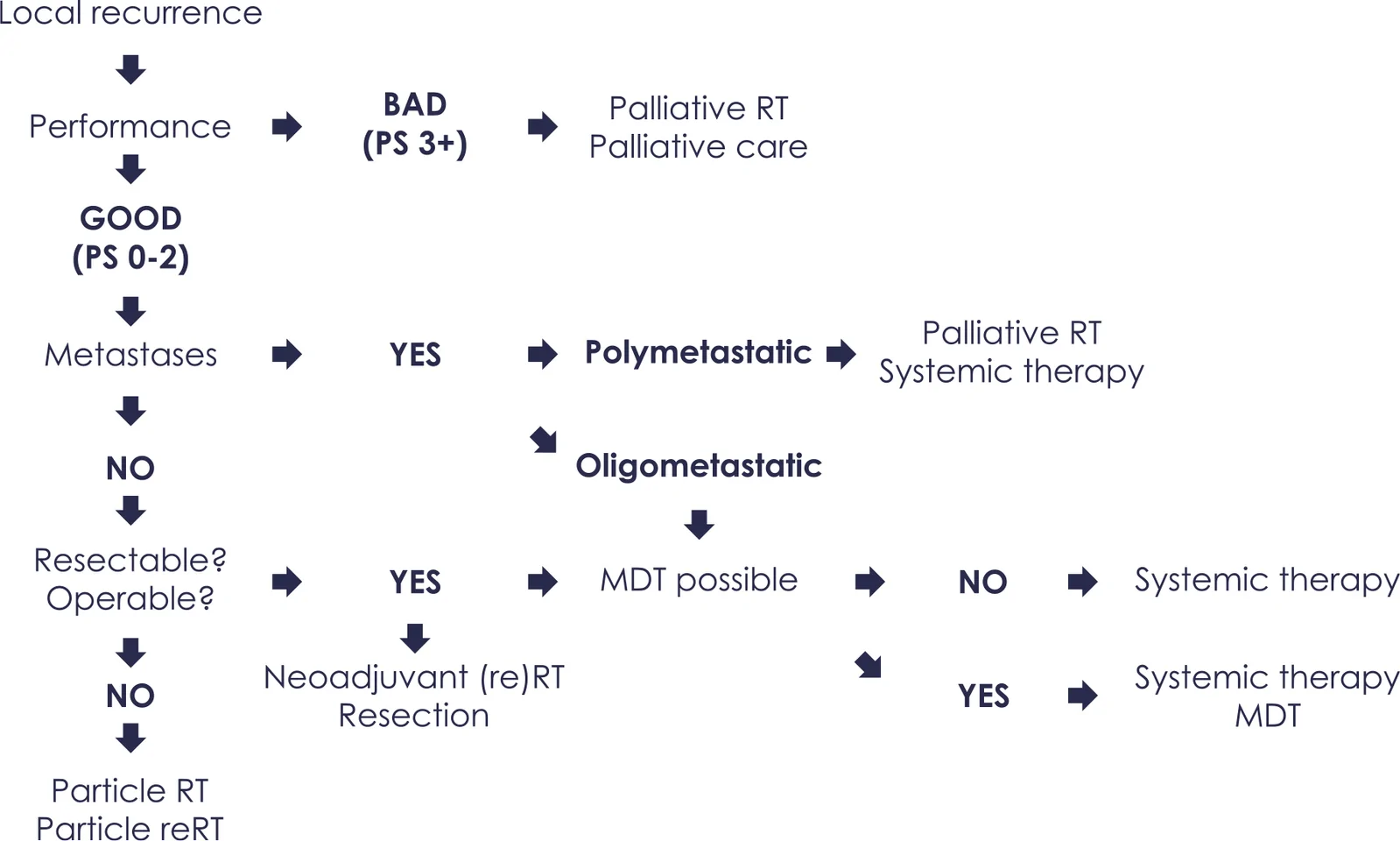
Patient selection for particle therapy of locally recurrent rectal cancer. MDT = metastasis-directed treatment; PS = performance status; reRT = reirradiation; RT = radiation therapy; SBRT = stereotactic body radiation therapy

The favourable dose distribution characteristics of particle beams and the potential for dose escalation would increase the chances of cure of macroscopic radioresistant disease without significantly increasing the rate of severe toxicity. Thus, particle beam therapy would present a curative alternative for surgery in patients with unresectable or inoperable locally recurrent rectal cancer.

As regards the selection of patients for each curative-intent RT modality, tumour size and shape may provide useful criteria. Some centres set an upper limit for particle therapy at tumour sizes above 15 x 15 cm, there is still a significant number of patients which are not candidates for SBRT due to the size of the recurrent tumours which would be considered treatable with particles.^54,58^ Thus, particle beams can offer curative treatment to patients with recurrent tumours too large or too complex-shaped for SBRT.

While patients with recurrent tumours abutting or invading the gastrointestinal and urinary tract are routinely counselled against ablative RT, case reports show that in select cases, even such patients may be treated curatively with the application of ablative dose if all reasonable measures are taken to mitigate the risk of late, severe, possibly life-threatening toxicities, including resecting bowel loops at risk of radiation-induced necrosis.^81,82^ However, it should be stressed that these cases are exceptions to the rule and that such decisions lie exclusively in the domain of an experienced, well-versed multidisciplinary team that operates in a high-volume comprehensive cancer centre.

An alternative mitigation strategy in the case of hollow organ abutment is the use of spacers, which displace the hollow organs from the high-dose area and thus both render the application of ablative dose possible and significantly reduce the risk of severe late toxicities.^83-85^ Though it should be noted that Shinoto *et al*. report that out of 224 patients in the J-CROS-1404 trial 12 patients developed grade 3 or more late toxicities and all 7 patients who developed pelvic abscesses had undergone spacer insertion before particle beam RT, so this method may have a risk of toxicities independent of ablative RT.^58^

A randomized trial between surgery and particle beam therapy for patients with unresectable locally recurrent rectal cancer is neither practicable nor is it acceptable from an ethical point of view, given that according to the literature, patients who were treated with an incomplete resection with macroscopic residual tumour (R2 resection) had at best comparable and at worst worse outcomes than those who had not been surgically treated and received only palliative / conservative treatment.^11-13^

Similarly, comparing the results of conformal photon radiation therapy and ablative radiation therapy (either SBRT or particle therapy) is meaningless, since their roles in the management of this diverse patient group are different: the former is an adjunct to surgical treatment while the latter, with the possibility of dose escalation, is an alternative for surgery when surgery is not possible.^61,63^ While retrospective studies have shown that certain patients are better candidates for one over the other, until we have more robust, prospective data, no definitive recommendations can be made for patient selection.

Particle therapy avoids mutilating surgical procedures in patients with posterior and lateral recurrences, in extensive recurrent tumours spanning more than one compartment, as well as offering the chance of cure for patients with unresectable disease and contraindications to surgery with acceptable toxicity.

The authors propose the following algorithm for the selection of candidates for particle therapy of locally recurrent rectal cancer:

In conclusion, for unresectable and inoperable locally recurrent rectal cancer ablative radiotherapy techniques such as SBRT and particle beam therapy offer a curative treatment approach. Further research is needed to stratify patients according to their need and eligibility for the different possible modalities of treatment.

Currently running prospective studies will furnish us with more robust results to facilitate patient stratification towards optimal modes of treatment as well as optimizing treatment protocols to achieve optimal results.

## Supplementary Material

Supplementary Material Details
