# Supplementary material for: The role of particle therapy in the treatment of locally recurrent rectal cancer: a systematic review
Source: Radiol Oncol. 2026 Sep 7;60(3):323–31. doi: 10.2478/raon-2026-0036 (PMC13548680; doi:10.2478/raon-2026-0036)
Supplement: Supplementary file 1 [file raon-2026-0036_sm.pdf]

The role of particle therapy in the treatment of locally recurrent rectal cancer: a systematic review

Ana Perpar, Petra Georg, Ajra Secerov Ermenc

doi: 10.2478/raon-2026-0036

SUPPLEMENTARY TABLE 1. The results of photon RT of locally recurrent rectal cancer.

Author	Year	# pts	mFU (months)	Techn	Median dose (Gy)	IORT	FRACT	CT	M1	Target size	Surgery	Local control (%, median in months)						Overall survival (%, median in months)					
												1Y	2Y	3Y	4Y	5Y	Median	1Y	2Y	3Y	4Y	5Y	Median
Al-Haidari[31]	2020	67		3DCRT	45	N	N	67,0%	25%	107 ccm	Y									32,0			15,6
	SURGERY<br>NO SURGERY																			62<br>16			39,2<br>8,9
Bosman[32]	2014	135	28	IMRT	25	100,0%	N	N	0%		Y											35,2	
Cai[33]	2015	71	31	IMRT	55	N	N	Y	32%		19,7%							80,1		36,5			29,0
	SURGERY																	92,3		53,3			
Chung[34]	2019	41	53,7	IMRT	50	N	N	N	12%	3,3 cm	21,9%		49,9				23,3		55,3				33,8
Jo[35]	2016	22	24,9	3DCRT	57,6	N	N	50,0%	0%		N	88,4	74,6					89,4	82,0				
Kishan[36]	2017	25	36,9	3DCRT	49,6	33,3%	N	92,0%	40%		64,0%			73,3	68,6	68,6				51,6	32,9	28,2	35,8
Park[37]	2019	25	21,5	3DCRT	45	N	N	56,0%	0%	191,1 ccm	44,0%		44,6	29,7					54,6	49,7			
Sun[38]	2012	72	24	3DCRT	36	N	Y	Y	0%	≤ 5 cm 34,7%<br>> 5 cm 65,3%	N			31,1			17,0			45,1			32,0
Susko[39]	2016	33	13,9	2D-3D	30	N	N	75,0%	0%		42,0%		15,7						47,8				23,1
	SURGERY<br>NO SURGERY																						32,3<br>13,3
Tanaka[40]	2017	32	16	3DCRT	60	N	N	N	56%	39 mm	N	51,5	24,5	19,6	19,6	13,1		82,6	56,5	45,2	38,7	23,2	
	HIGH BED<br>LOW BED																	100<br>77,9					
Tao[41]	2017	102	28	3DCRT	39	21,6%	Y	91,0%	YES[2]		45,0%		51,0	40,0					61,0	39,0			30,0
	SURGERY<br>NO SURGERY																			62<br>20			47<br>17
Watanabe[42]	2020	18	42	3DCRT	59,4	N	N	56,0%	0%	49 mm	N					34,8						54,4	

1Y = 1-year outcomes; 2D-3D = 2-dimensional and 3- dimensional conformal radiation therapy; 3DCRT = 3-dimensional conformal radiation therapy; BED = biologically effective dose; CT = chemotherapy; FRACT = altered fractionation regimes; IMRT = intensity-modulated radiation therapy; IORT = intraoperative RT; M1 = proportion of metastatic patients; mFU = median follow-up; Pts = patients; reRT = reirradiation; Tech = RT technique.

White background for studies with radiation-naïve cohort, blue background for studies with reirradiated cohort, orange background for mixed radiation-naïve and reirradiated cohort.

* The study only reports that metastatic patients were included in the analysis. It does not report how many were included.

SUPPLEMENTARY TABLE 2. Results of definitive RT of locally recurrent rectal cancer (stereotactic body radiation therapy [SBRT] and particle therapy)

Author	Year	# pts	mFU	Tech	Dose (med, Gy/fx)	Size	G3+	Local control (%, median in months)						Overall survival (%, median in months)					
								1Y	2Y	3Y	4Y	5Y	Median	1Y	2Y	3Y	4Y	5Y	Median
Dagoglu[48]	2015	18	38	SBRT	25/5	90,1 ccm	2 pts	100,0	93,7	85,9			38,0	76,8	65,9	59,3			40,0
DeFoe[49]	2011	14	16,5	SBRT	36/3/3	52,5 ccm	0%	90,9	68,3					90,0	78,8				
Johnstone[50]	2021	69	28	SBRT	30/5	13,4 ccm	NR								77,0				38,7
Kim[51]	2008	23	31	SBRT	39/3	26 ccm	1 pt				74,3						24,9	23,2	37,0
Minneci[52]	2021	23	91	SBRT	40/5	GTV 13,4 ccm	13%			95,6		82,6							
Smith[53]	2020	30	24,5	SBRT	30/5	14,3 ccm	1 pt	84,9	69,0					95,0	84,4				28,3
Yamada[54]	2016	186	42	C12	73,6/16	34 mm	2%	100,0								72,0		53,0	69,0
		10	42	C12	67,2							35,0				20,0		20,0	
		21	42	C12	70,4							77,0				53,0		26,0	
		155	42	C12	73,6							88,0				78,0		59,0	
Yamada[55]	2022	77	45	C12	70,4/16	40 mm	21%			69,0		62,0				61,0		38,0	47,0
Takagawa[56]	2024	10	28,7	p+	72/36	34 mm 26,1 ccm	10%	70,0	58,3					100,0	60,0				26,0
Takagawa[57]	2023	23	37,4	p+	70/35	37 mm	13%			55,0		47,2				72,1		44,6	54,4
Shinoto[58]	2019	224	62	C12	73,6/16	30 mm	5%			93,0		88,0				73,0		51,0	
Shiba[59]	2024	28	51,2	C12	73,6/16	44 mm	11%			88,0		83,0	NR			89,0		50,0	75,6
Koroulakis[60]	2021	28	28,6	p+	44,4/?[2]	86,4 ccm	14%	66,3						81,8					29,2
Jeans[61]	2023	86	49,2	IMRT	45/15	50 mm	10,5/15%		73,3			63,3			62,5			25,7	31,2
		85	92,4	C12	70,4/16	29 mm	14,1/0%		58,2			53,6			83,1			46,8	54,0
Hiroshima[62]	2021	13	42,9	p+	72/24	63 mm	0%			80,2						71,3			67,1
Chung[63]	2022	35	45,7	C12	70,4/16	25 mm	19/6%	93,3		87,3				97,0		86,4			
		31	22,8	IMRT	50/25	30 mm	13/0%	89,3		43,7				88,9		54,5			
Cai[64]	2020	25	19,6	C12	72/20	84,5 ccm	12%	90,4	71,8					82,9	65,1				
		9	19,6	C12	< 66			76,2	30,5					77,8	55,6				
		16	19,6	C12	≥ 66			100,0	100,0					86,5	69,2				
Cai[65]	2023	24	23,8	C12	72/20	GTV 57,1 ccm	12,5%	100,0	93,3					86,7	81,3				
Barcellini[66]	2020	14	18	C12	60/16	GTV 154 ccm	0%	78,0	52,0					100,0	76,2				

[1] 1Y = 1-year outcomes; C12 = carbon ion beam; G3+ - proportion of patients with grade 3 or above toxicities; GTV = gross target volume; IMRT = intensity-modulated radiation therapy; med = median; mFU = median follow-up in months; NR = not reported; p+ = proton beam; Pts = patients; reRT = reirradiation; SBRT = stereotactic body radiation therapy; Tech = RT technique

White background for studies with radiation-naïve cohort, blue background for studies with reirradiated cohort, orange background for mixed radiation-naïve and reirradiated cohort.

SUPPLEMENTARY TABLE 3. Ongoing studies on radiation therapy (RT) in locally recurrent rectal cancer

Country	Study Name / ID	Diagnosis	Beam type	ReRT	Outcome
Japan[69]	HIMAT 1351	locally recurrent rectal cancer	C12	NO	Primary: 3-year local control<br>Secondary: response rate, survival rate, progression-free survival, complication rate and pain response
Japan[70]	UMIN000022731	locally recurrent rectal cancer	C12	YES	Primary: 3-year local control
China[71]	SPHIC-TR-CRC2021-01<br>NCT05807984	locally recurrent rectal cancer	C12[1]	YES (> 1 course)	Primary: local control<br>Secondary: overall and progression-free survival
China[72]	TORCH-R	locally recurrent rectal cancer oligometastatic patients included	EBRT[2]<br>SBRT	BOTH	Primary: local objective response rate
S. Korea[73]	RECCPT	locally recurrent rectal cancer	p+[3]	YES	Primary: 3-year local control
USA[74]	IMPARC	locally recurrent rectal cancer	p+	YES	Primary: maximum tolerated dose (MTD) of hypofractionated IMPT (3 groups based on dose level)
Denmark[75]	ReRAD II	locally recurrent rectal cancer	p+[4]	YES	Primary operable: rate of pathological resection<br>Primary definitive: 1-year local control.<br>Secondary: local re-recurrence 6, 12 and 24 months after treatment, disease-free and overall survival, toxicity and quality of life.
Italy[76]	RETRY	locally recurrent rectal cancer oligometastatic patients included	ANY[5]	YES	Primary: local control
France[77]	GRECCAR-15	locally recurrent rectal cancer	EBRT[6]	YES	Primary: R0 resection rate

[1] 74 Gy in 20 fractions

[2] Primary RT to 25–40 Gy in 5 fractions, reirradiation to 15–30 Gy in 5 fractions, followed by 18 weeks of various regimes of chemotherapy and immunotherapy (toripalimab). Further management determined by multidisciplinary team.

[3] Proton therapy with simultaneous integrated boost technique to gross tumor volume to a dose of 70.4 Gy in 16 fractions and dose to clinical target volume 44.8 Gy in 16 fractions with Capecitabine in a salvage setting with or without surgery

[4] Neoadjuvant (55 Gy RBE à 1.25 Gy RBE twice daily) or definitive (57.5-65 Gy RBE à 1.25 Gy RBE twice daily)

[5] The protocol does not decidedly prescribe local treatment aside from it being reirradiation in a neoadjuvant context.

[6] Randomized phase III trial comparing neoadjuvant chemotherapy followed by pelvic RT (30.6 Gy with capecitabine) versus neoadjuvant chemotherapy alone

C12 = carbon ion beams, p+ = proton beams
